# Global Distribution of Fluoroquinolone and Colistin Resistance and Associated Resistance Markers in *Escherichia coli* of Swine Origin – A Systematic Review and Meta-Analysis

**DOI:** 10.3389/fmicb.2022.834793

**Published:** 2022-03-09

**Authors:** Shivdeep Singh Hayer, Alejandro Casanova-Higes, Eliana Paladino, Ehud Elnekave, Andre Nault, Timothy Johnson, Jeff Bender, Andres Perez, Julio Alvarez

**Affiliations:** ^1^Department of Veterinary Population Medicine, College of Veterinary Medicine, University of Minnesota-Twin Cities, Saint Paul, MN, United States; ^2^Department of Biology, College of Arts and Sciences, University of Nebraska Omaha, Omaha, NE, United States; ^3^Departamento de Patología Animal, Facultad de Veterinaria, Universidad de Zaragoza, Zaragoza, Spain; ^4^Koret School of Veterinary Medicine, The Robert H. Smith Faculty of Agriculture, Food and Environment, The Hebrew University of Jerusalem, Jerusalem, Israel; ^5^Health Science Libraries, University of Minnesota-Twin Cities, Saint Paul, MN, United States; ^6^Department of Veterinary and Biomedical Sciences, College of Veterinary Medicine, University of Minnesota Twin-Cities, Saint Paul, MN, United States; ^7^School of Public Health, University of Minnesota-Twin Cities, Saint Paul, MN, United States; ^8^VISAVET Health Surveillance Center, Universidad Complutense Madrid, Madrid, Spain; ^9^Department of Animal Health, Facultad de Veterinaria, Universidad Complutense Madrid, Madrid, Spain

**Keywords:** *Escherichia coli*, antimicrobial resistance, systematic review, pigs, fluoroquinolones, colistin, *mcr*, *qnr*

## Abstract

**Background:**

Fluoroquinolones and polymyxins (colistin) are considered as critical drugs for human medicine. Antimicrobials of these classes are also used in swine production worldwide and this usage can contribute to selection of antimicrobial resistance (AMR), which is a threat to both human and animal health. Given the dynamic epidemiology of AMR, updating our knowledge regarding distribution and trends in the proportion of resistant bacteria is of critical importance.

**Objectives:**

The aim of this systematic review and meta-analysis was to describe the global prevalence of phenotypic and genotypic resistance to fluoroquinolones and colistin in *Escherichia coli* collected from swine.

**Results:**

Four databases (PubMed, PubAg, Web of Science, and CAB abstracts) and reports of national surveillance programs were scanned and 360 articles were included in the analysis. We identified higher prevalence levels of fluoroquinolone and colistin resistance in isolates from pig populations in Asia compared to Europe. The heterogeneity of pooled estimates was also higher in Asian countries suggesting that prevalence of AMR is still not fully characterized. There was a major knowledge gap about the situation of AMR in South American and African countries. We also identified key deficiencies in how AMR data was reported in the studies. A meta-analysis using 6,167 publicly available genomes of swine *E. coli* established the prevalence and global distribution of genetic determinants that can lead to fluoroquinolone and colistin resistance.

**Conclusion:**

This study provides the most comprehensive information on prevalence of phenotypic and genotypic resistance to key antimicrobials in pig populations globally. There is a need to establish national surveillance programs and effective policies, particularly in certain world regions, to curtail the threat of evolution of resistant isolates in swine production that can potentially contribute to public health detrimentally.

## Introduction

The threat posed by the emergence of antimicrobial resistance (AMR) worldwide has led to an increased focus on the importance of monitoring both antimicrobial use (AMU) and AMR in public health. A high percentage of the total amount of antimicrobials produced worldwide are consumed by animals (Van Boeckel et al., 2015), which is a risk factor for selection of resistant bacteria that could impact both animal and public health. Monitoring AMR in animal production is essential for ensuring public health and can complement AMR surveillance in humans, and for this reason most national and regional AMR monitoring systems have an animal and/or animal-based food component. AMU is particularly common in more intensified animal production systems, such as poultry and swine (Krishnasamy et al., 2015). In these animal species antimicrobials have been used to promote growth, for prophylaxis, for metaphylaxis and as therapeutic agents against a multitude of infectious microorganisms (Barton, 2014), some of which can also cause foodborne zoonoses. Specifically, fluoroquinolones are used in food animal medicine, including swine medicine, globally (Nhung et al., 2016; Lekagul et al., 2019). Colistin is also used in swine medicine in a large number of countries (Rhouma et al., 2016) but colistin use has been the subject of increased restrictions (Wang et al., 2020).

Not all AMR are considered equally threatening for human and animal health and the World Health Organization (WHO) has classified certain antimicrobial classes as being critically important for human medicine, including fluoroquinolones and colistin (Collignon et al., 2009). Certain antimicrobials belonging to these classes, such as enrofloxacin, are used in swine production (Hallenberg et al., 2020; Hayer et al., 2020b; Cuong et al., 2021), even though their use in animals can potentially lead to cross-selection of genetic determinants of resistance against antimicrobials used in human medicine such as ciprofloxacin (Burow et al., 2018).

Traditionally, resistance to fluoroquinolones was thought to be mediated mainly by chromosomal mutations in quinolone resistance determining regions (QRDRs) in *gyrA*, *gyrB*, *parC*, and *parE* genes (Hayer et al., 2020a). In the last two decades however, plasmid-mediated genes conferring quinolone resistance (*qnr* genes) have been increasingly reported, making the genetic backbone of fluoroquinolone resistance even more complex (Jacoby et al., 2008; Poirel et al., 2012). Similarly, colistin resistance was thought to be conferred only by chromosomal mutations but the recent discovery of plasmid-mediated genes encoding colistin resistance (*mcr* genes) (Liu et al., 2016) provides another example of AMR as a constantly evolving threat, in addition to demonstrating of the usefulness of genomic epidemiology in AMR surveillance.

Because of the widespread interest in AMR among clinicians, veterinarians and public health practitioners, there has been a massive increase in the number of scientific publications focusing on this topic in recent years (Torres et al., 2021). For this reason, a systematic review was performed to compile data regarding the prevalence of phenotypic and genotypic resistance against two critically important antimicrobial classes (fluoroquinolones and colistin) for which plasmid-mediated resistance mechanisms have been increasingly reported recently in a widely used indicator bacteria such as *E. coli* isolated from swine populations, an important source of animal meat worldwide. The aims of this systematic review and meta-analysis were to (1) provide pooled prevalence estimates of phenotypic and genotypic resistance to fluoroquinolones and colistin in *E. coli* collected from healthy and diseased pigs and (2) identify common genomic traits of the bacterial isolates carrying *mcr*, *qnr* or chromosomal mutations in QRDRs.

## Methods

### Literature Search

Four databases (PubAg, Web of science, PubMed, and CAB abstracts) were selected to retrieve relevant abstracts. The exact search strings have been provided in Supplementary File 1. All the databases were initially screened on April 13, 2017 and updated on July 24, 2021. References were retrieved and imported to Zotero (version 5.0.96.3) and duplicate articles were removed. Additionally, reports including information on AMR in *E. coli* from swine not published in the peer-reviewed literature were recovered from the reports of national and international agencies coordinating surveillance programs for AMR (EFSA, DANMAP, MARAN, NORMVET, SVEDRES, FINRES, NARMS, RESAPATH, and CIPARS).

The relevance of the retrieved references (titles and abstracts when available) was first screened by determining if there was a mention to AMR, *Escherichia coli* or *Enterobacteriaceae*, and swine (or livestock in general). The full-texts of articles retained at this stage were then retrieved and subjected to a second screening in which relevant data was extracted (see below). Data was collected from articles fulfilling the following inclusion criteria:

1. There was information on prevalence of genotypic and/or phenotypic AMR to fluoroquinolones (ciprofloxacin, enrofloxacin) and/or colistin or polymyxin E, AND
2. Resistance was measured in *E. coli*, AND
3. Bacterial isolates were retrieved from pigs (healthy or diseased/dead).

Studies were excluded if the articles were written in a language other than English, if they provided no original information (review articles), if they were experimental in nature (drug trials under experimental conditions, manipulation of plasmids etc.), if information was not provided for *E. coli* or bacteria were not isolated from pigs, if a denominator (number of isolates tested) from which the resistant isolates were obtained was not provided, if bacterial isolates were not clearly classified as resistant/non-resistant, if isolates were recovered using selective media, if colistin resistance was measured by disk diffusion or if the articles were focused on antimicrobial usage without providing information on AMR.

### Data Extraction

Data from the selected articles was extracted using a predesigned datasheet in Microsoft Excel 2013 developed specifically for this review (Supplementary File 2). Data extracted included year of study, first author’s last name, country in which the study was conducted, age and health status of the studied pig population, sampling strategy, methodology used to assess bacterial antimicrobial susceptibility, antimicrobials tested, criteria for classifying bacteria as resistant/non-resistant, total number of isolates tested, number of isolates resistant against each antimicrobial, antimicrobial usage and farm husbandry characteristics.

### Meta-Analysis of Literature

In order to account for differences in epidemiological cut-offs and clinical breakpoints, number of ciprofloxacin resistant isolates were harmonized on a per article basis. First, the number of isolates with MIC values ≥ 1 μg/ml were re-classified as resistant when MIC distribution data was available in the articles.

If MIC distributions were not available, the number of resistant isolates were adjusted according to the method used by Van Boeckel et al. (2019). Information on the breakpoints used was extracted from articles. For dilution methods, the following harmonization formula [adapted from Van Boeckel et al. (2019)] was used:

Adjusted number of resistant isolates equals number of resistant isolates reported in the article multiplied by (number of isolates in reference distribution with MIC ≥ 1 μg/ml divided by number of isolates in reference distribution with MIC ≥ breakpoint applied in the article).

For diffusion methods, the following harmonization formula was used:

Adjusted number of resistant isolates equals number of resistant isolates reported in the article multiplied by (number of isolates in reference distribution with zone of diffusion ≤ 21 mm divided by number of isolates in reference distribution with zone of diffusion ≤ applied breakpoint in the article).

The reference MIC and inhibition zone diameter distributions for ciprofloxacin were downloaded from the official EUCAST website (EUCAST, 2021). Current CLSI breakpoints (≥1 μg/ml for dilution methods, ≤ 21 mm for disk diffusion) were extracted from CLSI M100-S31 (CLSI, 2021). If information on breakpoints used was not provided in an article, it was assumed that the authors used CLSI breakpoints available at the time of publication of that specific article because CLSI breakpoints are the most commonly used breakpoints to differentiate clinically susceptible and resistant bacteria in non-European countries (Van Boeckel et al., 2019). Harmonization was not needed for reclassifying enrofloxacin and colistin resistance because the breakpoints for enrofloxacin did not change over the years, and therefore all articles measuring enrofloxacin resistance used clinical (veterinary) breakpoints, and a breakpoint of 2 μg/ml was uniformly applied for classifying *E. coli* isolates as colistin resistant in the articles selected for data extraction.

Pooled prevalence estimates of fluoroquinolone (ciprofloxacin or enrofloxacin) and colistin resistant *E. coli* were calculated at the country level separately for isolates collected from healthy animals and diseased animals. Articles that reported resistance data on isolates retrieved from healthy and diseased animals together were not included in the meta-analysis but are still retained in Supplementary File 2. Data from articles which did not mention the health status of animals were included in the analysis of isolates from healthy pigs because these studies compared their results with those of other healthy swine populations; isolates in these studies were collected from farms instead of from diagnostic laboratory collections and these studies were not outbreak investigations. In the articles containing data for both ciprofloxacin and enrofloxacin resistance for the same isolates, only data for ciprofloxacin resistance was used due to the high degree of cross-resistance between these two antimicrobials. Proportions of resistance to each antimicrobial were first transformed using Freeman–Tukey double arcsine transformation (Freeman and Tukey, 1950), and pooled estimates were then calculated using a random effects inverse variance model and back-transforming the estimates using library *meta* version 4.9-6 in R version 4.0.3 (Schwarzer, 2021). Statistical heterogeneity of pooled estimates (I^2^) was also extracted from this output. Choropleth maps of pooled prevalence estimates were created using QGis version 3.30.3. Pooled prevalence estimates were also estimated for *mcr* and *qnr* genes at a continent level based on the results of PCR or WGS recorded in the articles.

Correlation between pooled resistance levels in isolates from healthy pigs, diseased pigs and antimicrobial usage were estimated using Spearman’s correlation coefficient in R version 4.0.3. Antimicrobial usage data was extracted from European Medicine Agency’s report on annual sales of antimicrobial agents in European countries (European Medicines Agency, 2020). It should be noted that antimicrobial usage reported was not sub-grouped at animal species level and was aggregated across all food animals.

### Genomic Meta-Analysis

Data available for *E. coli* isolates was downloaded from the Enterobase database (Zhou et al., 2020) (last accessed July 24, 2021). We identified 6,167 isolates of swine origin and downloaded the raw reads for 5,819 isolates based on accession numbers provided in the Enterobase database. Three hundred forty-eight isolates were downloaded directly from Enterobase as these isolates were available on this webserver but had not been submitted to NCBI Genbank yet, or were finished, high quality genomes with complete chromosomes and plasmids which did not require an assembly. Raw reads were trimmed using Trimmomatic version 0.39 (settings: sliding window mode; number of bases to average across, 4; average quality required, 20) (Bolger et al., 2014). Genomes were assembled from raw reads using Shovill version 1.0.9 (Seemann, 2021b). The assembly statistics are mentioned in Supplementary File 4. Resistance genes, plasmid replicon types and virulence factors were detected in all genomes (6,167 genomes assembled as described above or directly downloaded from Enterobase) using Resfinder, Plasmidfinder and Virulence Factor Database (VFDB) databases in Abricate version 1.0.1 (Carattoli et al., 2014; Liu et al., 2019; Bortolaia et al., 2020; Seemann, 2021a). Chromosomal mutations associated with the development of phenotypic resistance to all antimicrobial classes including fluoroquinolones and polymyxins (colistin) were detected using Pointfinder version 4.1 (Bortolaia et al., 2020).

Random Forest models were built to analyze which genomic features (plasmid replicons, virulence factors, resistance mechanisms to other antimicrobials classes) were more highly associated with the presence or absence of specific resistance genes or chromosomal mutation. Three separate random forest models were created using a particular gene or mutation (*qnrS1*, *mcr1.1*, and *gyrA*-S83L) as a dependent variable and the remaining 602 genomic features as independent variables. Independent variables were first tested for correlation (*R* > 0.99) using *caret* library in R version 4.0.3 (Kuhn et al., 2021), and only one of these correlated variables were retained for downstream analysis to decrease collinearity in the models. Further removal of correlated features (up to *R* > 0.80) did not improve the error rates of these models. These models were built using the quantile classifier approach for imbalanced data implemented in library *randomForestSRC* version 2.12.1 (Ishwaran and Kogalur, 2021) in R version 4.0.3. Nodes were split using the AUC split rule and performance of models was assessed using G-mean. These models were run for 12,000 trees with 25 variables used for splitting at each tree node. Models were first run on a training data set containing 65% of the original data. This data was selected using stratified random sampling without replacement so that the proportion of isolates with a positive value (i.e., presence of the resistance marker) for the dependent variable in the training data was the same as in the overall data (library *caTools* version 1.18.2 in R version 4.0.3) (Tuszynski, 2021). The remaining data set (35%) was used as testing data. Models ran on training data were used to predict the variable importance of genomic features in the testing data. We also estimated univariate, unadjusted odds ratio to reflect effect size and direction of the relationship for the top ten most important variables and plotted these estimates using *ggPlot2* version 3.3.5 (Wickham et al., 2021).

## Results

### a) Literature Review

#### Characteristics of Articles Included

**FIGURE 1:**
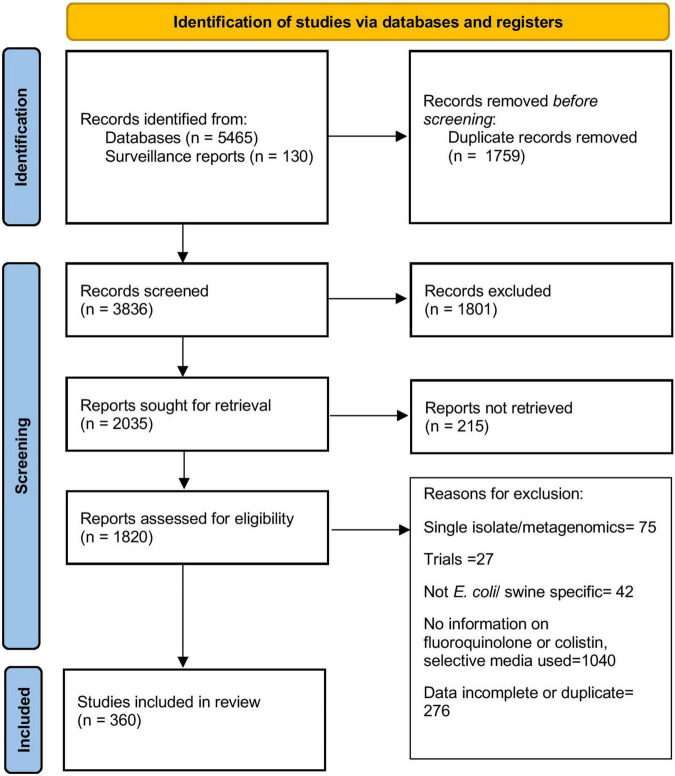
Flowchart describing selection of articles included in this review.

The steps in the literature search are summarized in Figure 1 and the characteristics of articles included in the final stage of the review (*n* = 360) are presented in Table 1. The studies included in the literature review were published between 1983 and 2021, with nearly 65% of them published post-2010. Articles contained information about isolates collected from as early as 1970 and as recent as 2020. Overall, information from 63 countries was retrieved. Most of the selected articles reported data from European (166 articles, 46.1%) or Asian (122 articles, 33.9%) countries. Phenotypic resistance data was available from 340 articles, obtained mostly through dilution (agar dilution or broth microdilution) (*n* = 218, 64.1%) and disk diffusion (*n* = 127, 37.4%) methods. In the 320 articles containing information about phenotypic fluoroquinolone resistance, 180 and 81 articles used the CLSI breakpoints and the guidelines issued by EUCAST to determine resistance, respectively, while the remaining used other criteria such as BSAC, DSKM, AFA, CA-SFM, breakpoints established by other regional surveillance programs, manufacturer’s guidelines, breakpoints from other articles or self-determined. Only six (1.87%) articles did not explicitly mention the interpretive criteria used to describe isolates as susceptible/resistant. Information on phenotypic colistin resistance was available in 106 articles. Since the interpretive criteria for colistin were introduced only in the last decade, use of interpretive criteria was not evaluated for colistin resistance.

**TABLE 1:** General characteristics of the data reported in the articles selected.

Characteristic	Specific feature	Number of articles
Phenotypic methods used (*n* = 340 articles)	Diffusion and dilution	6
	Dilution	212
	Diffusion	121
	Unknown	1
Genotypic methods (*n* = 57 articles)	PCR	52
	Whole genome sequencing	5
Publication Years (*n* = 360 articles)	before 2000	14
	2000–2005	47
	2006–2010	64
	2011–2015	86
	2016–2021	149
Time of sample collection (*n* = 360 articles)	Known	324
	Unknown	36
Age of pigs (*n* = 360 articles)	Described	235
	Partially described	7
	Unknown	118
Sample scheme (*n* = 360 articles)	Described	199
	Not described	161
Management (*n* = 360 articles)	Described	57
	Not described	303
Antibiotic use (*n* = 360 articles)	Mentioned	151
	Not mentioned	209
Continent (*n* = 360 articles)	Africa	9
	Asia	122
	Europe	166
	North America	39
	Oceania	7
	South America	17
Specific location/Geographic extent (*n* = 360 articles)	Described	323
	Not described	37
Health status (*n* = 360 articles)	Healthy	214
	Diseased	173
	Mixed	10
	Unknown	22

Health characteristics, exact geographic location or geographical scope of the study (e.g., national, regional) and year of sample collection were provided in 93.9% (*n* = 338), 89.7% (*n* = 323), and 90% (*n* = 324) articles, respectively. However, age of pigs, sampling scheme, history of antibiotic use at a farm or animal level and farm management characteristics were not specified in 32.7% (*n* = 118), 44.7% (*n* = 161), 58.1% (*n* = 209), and 84.2% (*n* = 303) articles, respectively.

#### Prevalence of Fluoroquinolone Resistance in Isolates From Healthy Pigs

**FIGURE 2:**
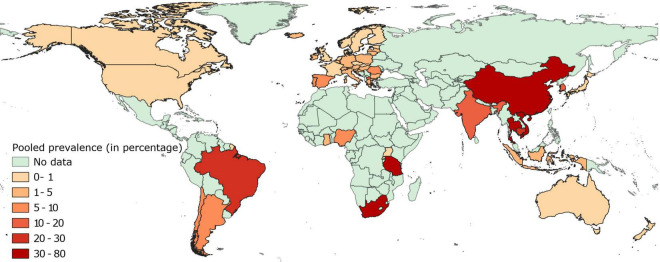
Global distribution of fluoroquinolone resistance in *E. coli* isolates collected from healthy pigs.

Pooled estimates of fluoroquinolone resistance have been provided in Supplementary File 2 and mapped in Figure 2. Briefly, data on fluoroquinolone resistance in isolates collected from healthy pigs was available for 55 countries, 32 of which were located in Europe. In this continent, prevalence of fluoroquinolone resistance in these isolates was below 1% in 11 countries and between 1 and 5% in 17 countries, while higher levels (9–14%) were reported in Spain, Portugal, Romania, and North Macedonia.

In Asia, prevalence of fluoroquinolone resistance was markedly higher, with only isolates from healthy pigs in Japan and Indonesia having fluoroquinolone-resistance levels close to 1%. Prevalence of fluoroquinolone resistant isolates in *E. coli* from healthy pigs in other Asian countries ranged between moderate-high (13–46%) in India, South Korea, the Philippines, Thailand, Vietnam, and China.

Similar to certain European countries, the prevalence of fluoroquinolone resistance in isolates from healthy pigs was extremely low (<1%) in Canada, United States, Australia, New Zealand, and Grenada. The prevalence of fluoroquinolone resistance in isolates from healthy pigs in Africa and South America was extremely variable, with resistance levels ranging between extremely low (<1% in Rwanda and Uganda), low (2–9% in Ghana, Nigeria, Argentina, and Chile), and high (21–38% in South Africa, Tanzania, and Brazil).

#### Prevalence of Colistin Resistance in Isolates From Healthy Pigs

**FIGURE 3:**
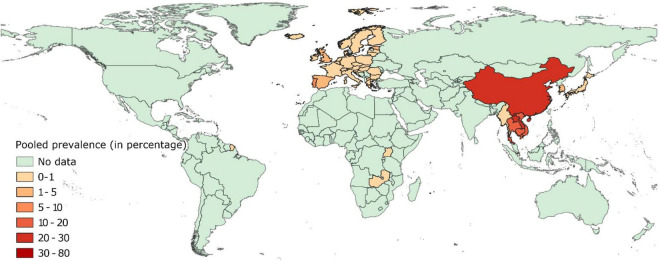
Global distribution of colistin resistance in *E. coli* isolates collected from healthy pigs.

Pooled estimates of colistin resistance have been provided in Supplementary File 2 and mapped in Figure 3. Data on colistin resistance from healthy pigs was available from 42 countries. Prevalence of colistin resistance was <1% in isolates from healthy pigs in 27 European countries and ranged between 1 and 6% in isolates from Greece, United Kingdom, Spain, Estonia, North Macedonia, and Portugal.

Prevalence of colistin resistance was higher in isolates from healthy pigs in Asia. Prevalence of colistin resistance in isolates from healthy pigs in Japan, South Korea, and Myanmar was <1%, whereas 10–23% of such isolates were colistin-resistant in Vietnam, Thailand, Cambodia, China, and Laos. Data from African and South American countries was scarce. Colistin resistance was < 1% in isolates from healthy pigs in Zambia and Uganda.

#### Statistical Heterogeneity of Pooled Prevalence Estimates

There were 64 pooled prevalence estimates of fluoroquinolone and colistin resistance in isolates from healthy pigs in European countries, and 45 (70.3%) of these estimates were statistically homogenous which indicates a relatively high reliability of these estimates (Supplementary File 2). In contrast, there were 33 such estimates available from non-European countries of which 14 (42.4%) were statistically heterogenous and in 13 cases (39.4%), heterogeneity was not estimated as data was only available from one article for certain combinations of country-antimicrobial-health status (Supplementary File 2).

#### Prevalence of Fluoroquinolone and Colistin Resistance in Isolates From Diseased Pigs

Pooled estimates of fluoroquinolone and colistin resistance in isolates from diseased pigs and associated statistical heterogeneity are summarized and mapped in Supplementary File 3 and detailed in Supplementary File 2. Briefly, the patterns of resistance to these antimicrobials were similar in isolates from healthy and diseased pigs and correlated moderately (*R* = 0.60 for fluoroquinolone resistance based on 31 countries, 0.45 for colistin resistance based on 14 countries, using Spearman’s rank correlation coefficient). However, the pooled prevalence estimates of fluoroquinolone and colistin resistances were 16.3 (95% CI = 4.44–28.2) and 14.7 (95% CI = 2.58–26.8) times higher, respectively in isolates from diseased pigs as compared to those from healthy pigs.

Prevalence of fluoroquinolone and colistin resistance in isolates from diseased pigs ranged between 0 and 10% in 33 and 8 non-Asian countries, respectively. Prevalence of fluoroquinolone resistance ranged between 10 and 30% in seven non-Asian countries and was even higher (36–57%) in Spain, Portugal, Romania, Brazil, and Argentina. Colistin resistance was also higher in isolates from diseased pigs in Germany (11.5%) and Spain (24%). The prevalence estimates of fluoroquinolone and colistin resistance in isolates from diseased pigs in Asia ranged between 5.7 and 70%.

#### Correlation Between Antimicrobial Use and Pooled Antimicrobial Resistance in European Countries

AMU and AMR data was available for fluoroquinolone and colistin in 19 and 8 countries, respectively. Correlation between fluoroquinolone usage and resistance was very strong (*R* = 0.85 in diseased pigs, 0.91 in healthy pigs). Correlation between colistin usage and resistance was very weak (*R* = 0.19- isolates from healthy pigs) and moderate (*R* = 0.59- isolates from diseased pigs).

#### Prevalence of *mcr* and *qnr* Genes Based on Data in the Articles Selected

Data on the prevalence of these genes was extremely scarce. For example, data on prevalence of *mcr* and *qnr* genes from isolates collected from healthy pigs was available for only 19 and 9 countries, respectively. Raw data and pooled prevalence of *mcr* and *qnr* genes are available in Supplementary Files 2, 4, respectively. *mcr-1* was the most common colistin resistance gene with pooled prevalence estimates ranging between 0.4–13% and 12–16% in isolates from healthy and diseased pigs, respectively depending on the continent. *qnrS* was by far the most prevalent *qnr* gene in isolates from Asia (12.8%-healthy pigs, 13.2%- diseased pigs). In contrast, *qnrB* was the most prevalent gene in North American isolates (2.54%- healthy pigs, 1.09%- diseased pigs) followed by *qnrS* (2.03%- healthy pigs).

### b) Genomic Meta-Analysis

#### General Characteristics of Genomes Available

Overall, 6,167 *E. coli* genomes of swine origin were available from Enterobase (last accessed July 24, 2021) (Supplementary File 5). Geographical information and time of sampling were available for 5,825 and 5,924 isolates, respectively. Isolates were collected between 1960 and 2021 from 54 countries. Isolates from North America (*n* = 3,526), Europe (*n* = 1,287), and Asia (*n* = 618) made up 88.1% of the total number of isolates. Since absolute prevalence cannot be estimated because of lack of metadata (number of isolates tested, whether selective media was used, health status of pigs), we were only able to estimate relative abundances of particular genes in comparison to other genes of the same family.

#### Relative Abundance of *mcr* and *qnr* Genes

**FIGURE 4:**
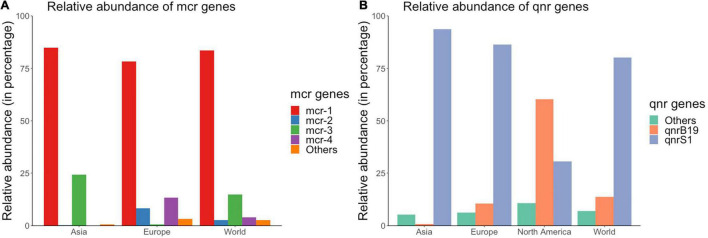
Relative abundances of (A) *mcr* and (B) *qnr* genes in publicly available *E. coli* genomes of swine origin.

There were 516 and 595 isolates that carried *mcr* and *qnr* genes, respectively. *mcr-1* was the most abundant *mcr* gene globally, with 83.5% of all *mcr*-positive isolates carrying this gene (Figure 4A). However, the second most prevalent *mcr* gene differed between European and Asian isolates (Figure 4A). In Europe, *mcr-4* (13.4%) and *mcr-2* (8.28%) were the second and third most abundant *mcr* gene; whereas in Asia, *mcr-3* (24.2%) was the second most prevalent *mcr* gene (Figure 4A). Only two *mcr-1* positive isolates were detected in 3,920 isolates from Africa, Oceania, South America, and North America.

*qnrS1* was the most prevalent *qnr* gene at the global level and was present in 80.2% of the 595 *qnr* positive isolates (Figure 4B). There were distinct geographical patterns in the relative abundance of *qnr* genes at a continent level (Figure 4B): *qnrS1* was the most prevalent gene in Asia and Europe (93.7% and 86.3% of all *qnr* positive isolates carried the *qnrS1* gene in each continent, respectively); while the most prevalent *qnr* gene in North American *qnr*-positive isolates was *qnrB19* (60.4%), and *qnrS1* was the second most prevalent gene (30.6%) (Figure 4B). *mcr-1* and *qnrS* genes were also the most prevalent genes globally based on the results of PCR/WGS available in the articles selected for this literature review (Supplementary Files 2, 4).

#### Characteristics of Isolates Carrying Mutations in Quinolone Resistance Determining Regions, *mcr* or *qnr* Genes

Chromosomal mutations in QRDRs, *mcr* or *qnr* genes were present in 1,626 isolates and these belonged to 384 different ST types. The top five sequence types (STs), clonal complexes, serotype, phylotype and *fimH*-type are presented in Table 2. Isolates of clonal complex 10 (ST10, ST48, and ST744) were the most frequent carriers of chromosomal mutations in QRDRs, *mcr* and *qnr* genes. Additionally, isolates of sequence type ST100, ST131, and ST206 were also frequent carriers of chromosomal mutations in QRDRs. Nearly 80% of the isolates were of phylotype A or B1, and only 6.77% were of phylotype B2 or D.

**TABLE 2:** Top five sequence type (ST), ST complexes, serotypes, phylotypes and *fim*H type of *E. coli* isolates carrying *mcr, qnr*, or chromosomal mutations in QRDRs (numbers in brackets represent the number of isolates carrying a particular genetic characteristic).

	ST	ST complex	Serotype	Phylotype	*fimH*
Chromosomal mutations in QRDRs (*n* = 1049)	100 (146), 10 (90), 744 (53), 131 (50), 206 (48)	10 (222), 165 (151), 206 (96), 23 (75), 131 (51)	O149:H10 (82), -:H10 (80), O89:H9 (40), O25:H4 (39), -:H4 (32)	A (556), B1 (236), C (72), B2 (52), D (41)	H54 (156), H24 (92), H23 (56), H31 (53), H41 (44)
*mcr* (*n* = 516)	10 (85), 48 (23), 101 (18), 1716 (17), 542 (10)	10 (163), 165 (25), 101 (25), 23 (20), 155 (13)	O130:H26 (17), O89:H9 (13), -:H9 (10), -:H10 (10), -:H4 (9)	A (315), B1 (128), D (21), C (19), E (11)	H54 (87), H2 (46), H2 (38), H31 (36), H41 (27)
*qnr* (*n* = 595)	10 (66), 48 (35), 515 (24), 58 (22), 1716 (19)	10 (176), 155 (32), 86 (23), 165 (21), 23 (20)	-:H11 (22), O130:H2 (18), -:H32 (17), -:H10 (17), -:H12 (14)	A (362), B1 (167), C (18), E (14), G (13)	H54 (91), H23 (58), H41 (44), H25 (32), H31 (30)

**FIGURE 5:**
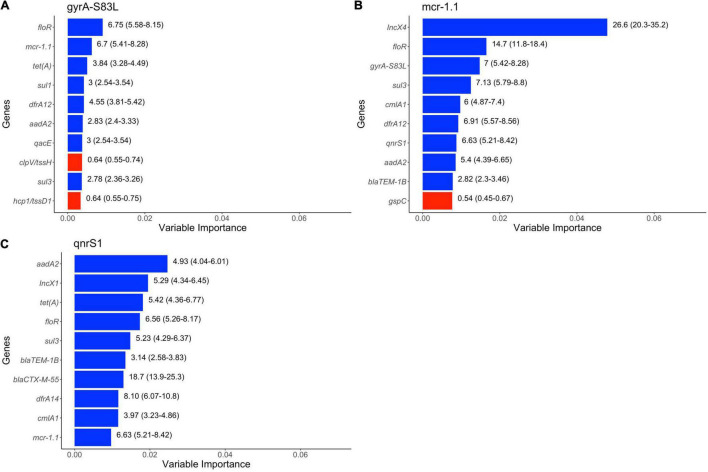
Top 10 variables of importance predictive of **(A)** *gyrA*-S83L mutation, **(B)** *mcr1.1* and **(C)** *qnrS1* in publicly available *E. coli* genomes of swine origin. Colors of bar represent positive (blue) and negative (red) association based on unadjusted odds ratio. The numbers on the right side of the bars represent unadjusted odds ratios (95% confidence intervals).

The results of random forests are presented in Figure 5. AMR genes, virulence genes and plasmid types were all found to be among the top ten variables of importance in predicting genes or mutations we were interested in. AMR genes which can confer resistance to phenicols (*floR*, *cmlA1*), tetracycline [*tet(A)*], sulfonamides (*sul1*, *sul3*), trimethoprim (*dfrA14, dfrA12*), penicillins (*bla*_TEM–1B_) and aminoglycosides (*aadA3*) were found to be top predictors of genes (*mcr1.1, qnrS1)* and chromosomal mutation in QRDR (*gyrA*-S83L). Disinfectant resistance gene (*qacE*) was also among the top predictors of *gyrA*-S83L mutation. Virulence genes (*tssH, tssD1*) and *gspC* were among the top predictors of *gyrA*-S83L mutation and *mcr1.1*, respectively, although these virulence genes were negatively associated with the presence of this mutation and gene. Presence of plasmid types IncX4 and IncX1 was also predictive of *mcr1.1* and *qnrS1*, respectively. Additionally, *bla*_CTX–M–55_, a gene that can confer resistance to critically important 3rd and 4th generation cephalosporins, was found to be predictive of *mcr1.1*. Finally, *mcr1.1, qnrS1* and chromosomal mutation (*gyrA*-S83L) were all predictive of each other’s presence.

## Discussion

The only other major review that is comparable to the scope of this work was authored by Van Boeckel et al. (2019). The current review complements the work of Van Boeckel et al. (2019) in several ways by including data from countries of all economic status, focusing on specific host (swine), bacteria (*E. coli*) and antimicrobials (fluoroquinolones and colistin), excluding isolates cultured on selective media which can artificially inflate the true prevalence of AMR, including isolates from both diseased and healthy animals and including genetic data. Additionally, Van Boeckel et al. (2019) selected only those articles that provided specific regional geographic location whereas we did not impose such restrictions in our inclusion criteria.

The pooled prevalence levels of fluoroquinolone and colistin resistance in isolates from healthy animals were low (<5%) in vast majority of the European, North American, and Oceanic countries. These countries have established several active surveillance programs to monitor the prevalence of AMR in isolates from livestock entering the food chain (e.g., NARMS, CIPARS, and SVARM). These surveillance programs can provide reliable estimates of the true prevalence of AMR in healthy animals based on stratified random sampling and can help to inform policy making. Pooled prevalence estimates of fluoroquinolone resistance were statistically homogenous for data from 19 European countries and Canada, suggesting that the estimates of AMR prevalence are more reliable in countries with established surveillance programs. Use of fluoroquinolones in swine agriculture has been restricted or banned in Australia (Abraham et al., 2015), Sweden (Lekagul et al., 2019), Denmark (Lekagul et al., 2019), and Netherlands (More, 2020). However, there were certain countries (Spain, Portugal, Romania, and North Macedonia) where the prevalence estimates of fluoroquinolone resistance were moderately high, and these prevalence levels correlated strongly with fluoroquinolone usage in food animals. These countries should monitor and regulate fluoroquinolone usage and prevalence of AMR in swine industry to further decrease resistance to this critical antimicrobial family. Similarly, some of these countries, such as Australia (Kidsley et al., 2018), Netherlands (More, 2020), Canada (Rhouma et al., 2019) and United States (Kumar et al., 2020), have also restricted the use of colistin to varying degrees in swine agriculture which might partly explain the low prevalence of colistin resistance in these countries.

Prevalence of fluoroquinolone and colistin resistance was consistently higher in isolates from Asia compared with Europe or America, with the exception of Japan. Animal agriculture sectors in these countries (specially China and India) are also among the highest consumers of antimicrobials in the world (Van Boeckel et al., 2015). There are several factors that can lead to an increased antimicrobial misuse in animal agriculture such as increased market incentives for faster growth, replacing good sanitary practices with AMU, easy availability of antimicrobials over-the-counter and lack of effective regulations of antimicrobials (Brower et al., 2017; Coyne et al., 2019). However, some countries are making significant policy changes to tackle the problem of AMR in farm animals. China, as an example, has banned the use of colistin as a growth promoter in animal agriculture since 2017 (Wang et al., 2020). The pooled prevalence estimates from these countries should also be interpreted with caution as there was significant statistical heterogeneity and pooled estimates were based on several regional studies with unspecified sampling strategies as opposed to well established national surveillance programs. There is an immediate need to promote the use of antimicrobial alternatives to maintain production levels, implement effective policies on AMU and set up regional and national surveillance programs in these countries to provide accurate estimates of AMR prevalence in animal agriculture in the region.

There were very few articles published from South American and African countries. Data from these countries is too scarce to derive solid conclusions on the status of AMR. The lack of availability of data on AMR might not be specific to swine agriculture: Torres et al. (2021) conducted a bibliographic analysis on trends in reports on AMR in food animals and determined that these countries have always lagged behind in publishing on this scientific topic. There is a clear need to establish AMR surveillance programs including those targeting food animals in these countries. It should be noted that the pig populations in these continents are scarcer as compared to Europe, North America, and South-East Asia, with a few exceptions such as certain regions in Brazil and Argentina (Robinson et al., 2014).

According to our results, clinical isolates were more resistant to fluoroquinolones and colistin compared to isolates from healthy animals. Some authors have suggested that treatment of diseased animals before sample collection may lead to higher levels of AMR compared to healthy populations (Aarestrup, 2005). Both fluoroquinolones and colistin are used as therapeutic options for various disease conditions in swine (Prescott, 2008; Kempf et al., 2016; Hayer et al., 2020b) and the higher likelihood of resistance in isolates from clinical submissions may be related to this hypothesis. Although AMR in clinical isolates is likely not representative of the levels of resistance in general swine populations, surveillance of AMR in clinical isolates can still be useful for monitoring purposes: first, in the absence of organized active surveillance programs, clinical submissions can be used as a passive surveillance system for monitoring levels of AMR, particularly against therapeutic agents such as fluoroquinolones and colistin. Such kind of surveillance can be particularly useful in resource limited settings where diagnostic laboratories can play dual roles, contributing to AMR surveillance while conducting diagnostic work. Second, clinical isolates can act as an early indicator of emerging resistance phenotypic and genotypic patterns which can potentially become endemic in healthy populations (Mather et al., 2016). The fact that the pooled prevalence estimates of resistance in isolates from diseased animals correlated well with antimicrobial consumption in this study provides further evidence that this information can serve as a proxy in monitoring effects of changes in antimicrobial usage on AMR.

Colistin resistance mediated by chromosomal mutations was considered to spread slowly *via* vertical transmission and with a slow pace of evolution, but the emergence of plasmid-borne *mcr* genes in mid-2010s ignited the concerns of a rapid spread of this resistance (Liu et al., 2016). The results from this genomic analysis provides further evidence of the rapid spread of plasmid-borne *mcr-1* genes in swine populations globally. *mcr-1* gene has been described to be borne on IncHI2, IncX4, and IncI2 plasmids (Wu et al., 2018). In the random forest models, plasmid type IncX4 was highly associated with *mcr1.1* gene carriage. This plasmid type can confer fitness advantage on host bacteria and can outcompete other plasmids (Wu et al., 2018), which is worrisome as these plasmids can disseminate easily with or without antimicrobial selective pressure. These results also provide a baseline for the distribution of other currently less frequent *mcr* genes and can be used for comparisons in the future.

*qnrS1* was the most prevalent gene in *E. coli* from swine populations, which differs from human populations wherein *qnrB* is more prevalent (Poirel et al., 2012; Jacoby et al., 2014). Widespread prevalence of *qnrS1* increases the challenge in controlling fluoroquinolone resistance. Firstly, this gene when combined with chromosomal mutations in QRDRs confer a fitness advantage to *E. coli* (Machuca et al., 2014). Secondly, these genes are commonly harbored on IncX1 plasmids which have the capability to disseminate AMR genes across different animal species and environment (Dobiasova and Dolejska, 2016; Slettemeås et al., 2019).

*mcr*, *qnr* and mutations in QRDRs were most commonly present in *E. coli* isolates belonging to clonal complex 10 (ST10, ST48, and ST744). Isolates of this clonal complex are known for their wide global dissemination and ease of crossing human and animal species. ST10 was the third most commonly reported extraintestinal pathogenic *E. coli* in a systematic review of human studies (Manges et al., 2019). Although less pathogenic and not as commonly prevalent in humans as ST10, ST744 has been also found in multiple animal species across the world and are known to carry and spread resistance genes to critical antimicrobials (Hasan et al., 2012; Hasman et al., 2015; Hayer et al., 2020a; Zingali et al., 2020). ST131 was also a common fluoroquinolone-resistant ST in swine populations in our analysis. This ST is perhaps the most widely studied and successful *E. coli* population due to its highly pathogenic potential and is often co-resistant to cephalosporins and fluoroquinolones (Price et al., 2013; Can et al., 2016; Manges et al., 2019). ST131 has been often regarded as a community-acquired pathogen adapted to humans but its origin and the role of animals in its spread is still unclear (Manges, 2019). Although rarely isolated from animals, a few studies have suggested that food animals might be a reservoir of certain ST131 lineages (Liu et al., 2018; Reid et al., 2019; Flament-Simon et al., 2020; Bonnet et al., 2021). Our analysis reinforces the need to further investigate the role of food animals including swine in dissemination and evolution of this medically important *E. coli* subtype.

The results of random forest models also suggest that key fluoroquinolone- and colistin-resistance mechanisms co-existed with several other genes which can confer resistance to antimicrobials other than fluoroquinolones or colistin. The presence of several AMR genes in the populations of same bacterial species can lead to phenotypic co-resistance to multiple antimicrobial classes (Cantón and Ruiz-Garbajosa, 2011). Co-resistance can further complicate the fight against AMR as use of one antimicrobial class can lead to co-selection of resistance to an unrelated antimicrobial class. This can have an impact on success or failure of a new policy implementation. For example, co-selection has been cited as a possible reason for sustenance of fluoroquinolone-resistance in commensal *E. coli* isolates collected in France between 2011 and 2018 despite a decrease in use of this antimicrobial class during the concurrent timeframe (Perrin-Guyomard et al., 2020).

Several limitations of this study must be kept in mind when interpreting the results. First, only articles written in English were included in this systematic literature review, thus potentially leading to language bias and the exclusion of data from certain countries. The purpose of this study was to obtain estimates on the frequency of resistance against fluoroquinolones and colistin in *E. coli* of swine origin, and therefore articles which focused on single new/unusual genes, description of single plasmids or case reports or a single farm/outbreak were excluded. As a result, the presence of certain genes encoding resistance to the antimicrobials of interest in this review may have been missed if they were only reported rarely. Lastly, key metadata entries such as age of the animal, antimicrobial usage and husbandry techniques employed were missing from the majority of the articles. Even when available, data on antimicrobial usage and husbandry was not detailed enough to be incorporated statistically in the meta-analytic models. Authors are encouraged to follow guidelines provided by EFSA on reporting antimicrobial studies (European Food Safety Authority et al., 2021), so that the information from their studies can be contextualized properly, particularly in case of low-resource, high AMR countries.

There are key limitations in the genomic meta-analysis as well. Random forest models provide possible associations between several genes but whether these genes were present on the same plasmid needs to be confirmed (e.g., using long-read sequencing, analysis of contigs, plasmid MLST etc.). The results of genomic analysis were dependent on existing databases of AMR, virulence and plasmid replicon genes. These databases are regularly updated depending on discovery of new genes and changes in gene nomenclature. Hence, the results might vary if updated databases are used on the same isolates.

## Conclusion

The aim of this systematic review and meta-analysis was to survey the global prevalence of resistance against specific key antimicrobials in indicator bacteria collected from swine. In general, the levels of fluoroquinolone and colistin resistance were higher in isolates from Asian countries compared to European and North American countries. There was also a severe paucity of data from African and South American countries in terms of both scientific literature and availability of bacterial genomes. A wide repertoire of genes responsible for fluoroquinolone and colistin resistance with some geographical variations in prevalence were identified in swine *E. coli* isolates. This review can serve as a useful baseline for both scientists and policy makers in understanding the current status of resistance to key critical antimicrobials in swine production globally.

## Author Contributions

SH, AP, and JA conceived this article. SH, AC-H, and EP collected the data. SH and AN devised the search strings for retrieving articles. SH, EE, AP, and JA analyzed the data. SH, EE, TJ, JB, AP, and JA contributed to writing the manuscript. All authors contributed to the article and approved the submitted version.

## Conflict of Interest

The authors declare that the research was conducted in the absence of any commercial or financial relationships that could be construed as a potential conflict of interest.

## Publisher’s Note

All claims expressed in this article are solely those of the authors and do not necessarily represent those of their affiliated organizations, or those of the publisher, the editors and the reviewers. Any product that may be evaluated in this article, or claim that may be made by its manufacturer, is not guaranteed or endorsed by the publisher.

## Supplementary Material

Supplementary File 1 Database search strings.

Supplementary File 2 Raw data collected from articles and detailed pooled prevalence estimates of phenotypic fluoroquinolone and colistin resistance.

Supplementary File 3 Pooled prevalence estimates of fluoroquinolone and colistin resistance in diseased pigs.

Supplementary File 4 Prevalence of mcr and qnr genes in articles selected for the literature review.

Supplementary File 5 Genotypic data including metadata from Enterobase, virulence genes, plasmid replicon types and resistance genes.

## References

[B1] Aarestrup F. M. (2005). Veterinary drug usage and antimicrobial resistance in bacteria of animal origin. *Basic Clin. Pharmacol. Toxicol.* 96 271–281. 10.1111/j.1742-7843.2005.pto960401.x 15755309

[B2] Abraham S. Jordan D. Wong H. S. Johnson J. R. Toleman M. A. Wakeham D. L. (2015). First detection of extended-spectrum cephalosporin- and fluoroquinolone-resistant *Escherichia coli* in Australian food-producing animals. *J. Glob. Antimicrob. Resist.* 3 273–277. 10.1016/j.jgar.2015.08.002 27842872

[B3] Barton M. D. (2014). Impact of antibiotic use in the swine industry. *Curr. Opin. Microbiol.* 19 9–15. 10.1016/j.mib.2014.05.017 24959754

[B4] Bolger A. M. Lohse M. Usadel B. (2014). Trimmomatic: a flexible trimmer for Illumina sequence data. *Bioinformatics* 30 2114–2120. 10.1093/bioinformatics/btu170 24695404 PMC4103590

[B5] Bonnet R. Beyrouthy R. Haenni M. Nicolas-Chanoine M.-H. Dalmasso G. Madec J.-Y. (2021). Host Colonization as a Major Evolutionary Force Favoring the Diversity and the Emergence of the Worldwide Multidrug-Resistant *Escherichia coli* ST131. *mBio* 12:21. 10.1128/mBio.01451-21 34425698 PMC8406181

[B6] Bortolaia V. Kaas R. S. Ruppe E. Roberts M. C. Schwarz S. Cattoir V. (2020). ResFinder 4.0 for predictions of phenotypes from genotypes. *J. Antimicrob. Chemother.* 75 3491–3500. 10.1093/jac/dkaa345 32780112 PMC7662176

[B7] Brower C. H. Mandal S. Hayer S. Sran M. Zehra A. Patel S. J. (2017). The Prevalence of Extended-Spectrum Beta-Lactamase-Producing Multidrug-Resistant *Escherichia Coli* in Poultry Chickens and Variation According to Farming Practices in Punjab, India. *Environ. Health Perspect.* 125:077015. 10.1289/EHP292 28749780 PMC5744676

[B8] Burow E. Grobbel M. Tenhagen B.-A. Simoneit C. Ladwig M. Szabó I. (2018). Antimicrobial susceptibility in faecal *Escherichia coli* from pigs after enrofloxacin administration in an experimental environment. *Berl. Münch Tierärztl Wochensch* 131 170–181. 10.2376/0005-9366-17079 35082672

[B9] Can F. Kurt-Azap Ö Ispir P. Nurtop E. Seref C. Loçlar I. (2016). The clinical impact of ST131 H30-Rx subclone in urinary tract infections due to multidrug-resistant *Escherichia coli*. *J. Glob. Antimicrob. Resist.* 4 49–52. 10.1016/j.jgar.2015.10.006 27436393

[B10] Cantón R. Ruiz-Garbajosa P. (2011). Co-resistance: an opportunity for the bacteria and resistance genes. *Curr. Opin. Pharmacol.* 11 477–485. 10.1016/j.coph.2011.07.007 21840259

[B11] Carattoli A. Zankari E. García-Fernández A. Voldby Larsen M. Lund O. Villa L. (2014). In Silico Detection and Typing of Plasmids using PlasmidFinder and Plasmid Multilocus Sequence Typing. *Antimicrob. Agents Chemother.* 58 3895–3903. 10.1128/AAC.02412-14 24777092 PMC4068535

[B12] CLSI (2021). *CLSI M100-ED31:2021 Performance Standards for Antimicrobial Susceptibility Testing, 31st Edition.* Wayne, PA: CLSI.

[B13] Collignon P. Powers J. H. Chiller T. M. Aidara-Kane A. Aarestrup F. M. (2009). World Health Organization Ranking of Antimicrobials According to Their Importance in Human Medicine: A Critical Step for Developing Risk Management Strategies for the Use of Antimicrobials in Food Production Animals. *Clin. Infect. Dis.* 49 132–141. 10.1086/599374 19489713

[B14] Coyne L. Arief R. Benigno C. Giang V. N. Huong L. Q. Jeamsripong S. (2019). Characterizing Antimicrobial Use in the Livestock Sector in Three South East Asian Countries (Indonesia, Thailand, and Vietnam). *Antibiotics* 8:33. 10.3390/antibiotics8010033 30934638 PMC6466601

[B15] Cuong N. V. Ly N. P. C. Van N. T. B. Phu D. H. Kiet B. T. Hien V. B. (2021). Feasibility study of a field survey to measure antimicrobial usage in humans and animals in the Mekong Delta region of Vietnam. *JAC Antimicrob. Resist.* 3:dlab107. 10.1093/jacamr/dlab107 34396120 PMC8360299

[B16] Dobiasova H. Dolejska M. (2016). Prevalence and diversity of IncX plasmids carrying fluoroquinolone and β-lactam resistance genes in *Escherichia coli* originating from diverse sources and geographical areas. *J. Antimicrob. Chemother.* 71 2118–2124. 10.1093/jac/dkw144 27165784

[B17] EUCAST (2021). *MIC and zone distributions and ECOFFs.* Available online at: https://www.eucast.org/mic_distributions_and_ecoffs/ (accessed November 29, 2021)

[B18] European Food Safety Authority Amore G. Beloeil P. Bocca V. Fierro R. G. (2021). Antimicrobial resistance guidance for 2021 data reporting. *EFS* 3:18. 10.2903/sp.efsa.2021.EN-6653

[B19] European Medicines Agency (2020). *Sales of veterinary antimicrobial agents in 31 European countries in 2018.* Amsterdam: European Medicines Agency, 104.

[B20] Flament-Simon S.-C. de Toro M. Mora A. García V. García-Meniño I. Díaz-Jiménez D. (2020). Whole Genome Sequencing and Characteristics of mcr-1–Harboring Plasmids of Porcine *Escherichia coli* Isolates Belonging to the High-Risk Clone O25b:H4-ST131 Clade B. *Front. Microbiol.* 11:387. 10.3389/fmicb.2020.00387 32265859 PMC7105644

[B21] Freeman M. F. Tukey J. W. (1950). Transformations Related to the Angular and the Square Root. *Ann. Mathemat. Statist.* 21 607–611. 10.1214/aoms/1177729756

[B22] Hallenberg G. S. Jiwakanon J. Angkititrakul S. Kang-air S. Osbjer K. Lunha K. (2020). Antibiotic use in pig farms at different levels of intensification—Farmers’ practices in northeastern Thailand. *PLoS One* 15:e0243099. 10.1371/journal.pone.0243099 33306684 PMC7732346

[B23] Hasan B. Sandegren L. Melhus Å Drobni M. Hernandez J. Waldenström J. (2012). Antimicrobial Drug–Resistant *Escherichia coli* in Wild Birds and Free-range Poultry, Bangladesh. *Emerg. Infect. Dis.* 18 2055–2058. 10.3201/eid1812.120513 23171693 PMC3557866

[B24] Hasman H. Hammerum A. M. Hansen F. Hendriksen R. S. Olesen B. Agersø Y. (2015). Detection of mcr-1 encoding plasmid-mediated colistin-resistant *escherichia coli* isolates from human bloodstream infection and imported chicken meat, denmark 2015. *Eurosurveillance* 20 1–5. 10.2807/1560-7917.ES.2015.20.49.30085 26676364

[B25] Hayer S. S. Lim S. H. Hong S. Elnekave E. Johnson T. (2020a). Genetic determinants of resistance to extended-spectrum cephalosporin and fluoroquinolone in *Escherichia coli* isolated from diseased pigs in the United States. *mSphere* 5:20. 10.1128/mSphere.00990-20 33115839 PMC8534314

[B26] Hayer S. S. Rovira A. Olsen K. Johnson T. J. Vannucci F. Rendahl A. (2020b). Prevalence and trend analysis of antimicrobial resistance in clinical *Escherichia coli* isolates collected from diseased pigs in the USA between 2006 and 2016. *Transboundary Emerg. Dis.* 67 1930–1941. 10.1111/tbed.13528 32097517

[B27] Ishwaran H. Kogalur U. (2021). *randomForestSRC-Fast Unified Random Forests for Survival, Regression, and Classification (RF-SRC).* Vienna: R Core Team.

[B28] Jacoby G. A. Strahilevitz J. Hooper D. C. (2014). Plasmid-mediated quinolone resistance. *Microbiol. Spectr.* 2:2013. 10.1128/microbiolspec.PLAS-0006-2013 PMC4288778 25584197

[B29] Jacoby G. Cattoir V. Hooper D. Martínez-Martínez L. Nordmann P. Pascual A. (2008). qnr Gene Nomenclature. *Antimicrob. Agents Chemother.* 52 2297–2299. 10.1128/AAC.00147-08 18426894 PMC2443900

[B30] Kempf I. Jouy E. Chauvin C. (2016). Colistin use and colistin resistance in bacteria from animals. *Int. J. Antimicrob. Agents* 48 598–606. 10.1016/j.ijantimicag.2016.09.016 27836380

[B31] Kidsley A. K. Abraham S. Bell J. M. O’Dea M. Laird T. J. Jordan D. (2018). Antimicrobial Susceptibility of *Escherichia coli* and *Salmonella* spp. Isolates From Healthy Pigs in Australia: Results of a Pilot National Survey. *Front. Microbiol.* 9:01207. 10.3389/fmicb.2018.01207 30038598 PMC6047343

[B32] Krishnasamy V. Otte J. Silbergeld E. (2015). Antimicrobial use in Chinese swine and broiler poultry production. *Antimicrob. Resist. Infect. Control* 4 1–9. 10.1186/s13756-015-0050-y 25922664 PMC4412119

[B33] Kuhn M. Wing J. Weston S. Williams A. Keefer C. Engelhardt A. (2021). *caret-Classification and Regression Training.* Vienna: R Core Team.

[B34] Kumar H. Chen B.-H. Kuca K. Nepovimova E. Kaushal A. Nagraik R. (2020). Understanding of Colistin Usage in Food Animals and Available Detection Techniques: A Review. *Animals* 10:1892. 10.3390/ani10101892 33081121 PMC7602861

[B35] Lekagul A. Tangcharoensathien V. Yeung S. (2019). Patterns of antibiotic use in global pig production: A systematic review. *Vet. Anim. Sci.* 7:100058. 10.1016/j.vas.2019.100058 32734079 PMC7386699

[B36] Liu B. Zheng D. Jin Q. Chen L. Yang J. (2019). VFDB 2019: a comparative pathogenomic platform with an interactive web interface. *Nucleic Acids Res.* 47 D687–D692. 10.1093/nar/gky1080 30395255 PMC6324032

[B37] Liu C. M. Stegger M. Aziz M. Johnson T. J. Waits K. Nordstrom L. (2018). *Escherichia coli* ST131- *H* 22 as a Foodborne Uropathogen. *mBio* 9:18. 10.1128/mBio.00470-18 30154256 PMC6113624

[B38] Liu Y. Y. Wang Y. Walsh T. R. Yi L. X. Zhang R. Spencer J. (2016). Emergence of plasmid-mediated colistin resistance mechanism MCR-1 in animals and human beings in China: A microbiological and molecular biological study. *Lancet Infect. Dis.* 16 161–168. 10.1016/S1473-3099(15)00424-7 26603172

[B39] Machuca J. Briales A. Labrador G. Díaz-de-Alba P. López-Rojas R. Docobo-Pérez F. (2014). Interplay between plasmid-mediated and chromosomal-mediated fluoroquinolone resistance and bacterial fitness in *Escherichia coli*. *J. Antimicrob. Chemother.* 69 3203–3215. 10.1093/jac/dku308 25139837

[B40] Manges A. R. (2019). *Escherichia coli* causing bloodstream and other extraintestinal infections: tracking the next pandemic. *Lancet Infect. Dis.* 19 1269–1270. 10.1016/S1473-3099(19)30538-9 31653525

[B41] Manges A. R. Geum H. M. Guo A. Edens T. J. Fibke C. D. Pitout J. D. D. (2019). Global Extraintestinal Pathogenic *Escherichia coli* (ExPEC) Lineages. *Clin. Microbiol. Rev.* 32 e135–e118. 10.1128/CMR.00135-18 PMC6589867 31189557

[B42] Mather A. E. Reeve R. Mellor D. J. Matthews L. Reid-Smith R. J. Dutil L. (2016). Detection of rare antimicrobial resistance profiles by active and passive surveillance approaches. *PLoS One* 11:1–12. 10.1371/journal.pone.0158515 27391966 PMC4938605

[B43] More S. J. (2020). European perspectives on efforts to reduce antimicrobial usage in food animal production. *Ir. Vet. J.* 73:2. 10.1186/s13620-019-0154-4 32002180 PMC6986017

[B44] Nhung N. T. Cuong N. V. Thwaites G. Carrique-Mas J. (2016). Antimicrobial Usage and Antimicrobial Resistance in Animal Production in Southeast Asia: A Review. *Antibiotics* 5:37. 10.3390/antibiotics5040037 27827853 PMC5187518

[B45] Perrin-Guyomard A. Jouy E. Urban D. Chauvin C. Granier S. A. Mourand G. (2020). Decrease in fluoroquinolone use in French poultry and pig production and changes in resistance among E. coli and Campylobacter. *Vet. Microbiol.* 243:108637. 10.1016/j.vetmic.2020.108637 32273016

[B46] Poirel L. Cattoir V. Nordmann P. (2012). Plasmid-Mediated Quinolone Resistance; Interactions between Human, Animal, and Environmental Ecologies. *Front. Microbiol.* 3:24. 10.3389/fmicb.2012.00024 22347217 PMC3270319

[B47] Prescott J. F. (2008). Antimicrobial use in food and companion animals. *Anim. Health Res. Rev.* 9 127–133. 10.1017/S1466252308001473 18983721

[B48] Price L. B. Johnson J. R. Aziz M. Clabots C. Johnston B. Tchesnokova V. (2013). The Epidemic of Extended-Spectrum-β-Lactamase-Producing *Escherichia coli* ST131 Is Driven by a Single Highly Pathogenic Subclone, *H* 30-Rx. *mBio* 4:13. 10.1128/mBio.00377-13 24345742 PMC3870262

[B49] Reid C. J. McKinnon J. Djordjevic S. P. (2019). Clonal ST131-H22 *Escherichia coli* strains from a healthy pig and a human urinary tract infection carry highly similar resistance and virulence plasmids. *Microb. Genom.* 5:000295. 10.1099/mgen.0.000295 31526455 PMC6807379

[B50] Rhouma M. Beaudry F. Letellier A. (2016). Resistance to colistin: what is the fate for this antibiotic in pig production? *Int. J. Antimicrob. Agents* 48 119–126. 10.1016/j.ijantimicag.2016.04.008 27234675

[B51] Rhouma M. Thériault W. Rabhi N. Duchaine C. Quessy S. Fravalo P. (2019). First identification of mcr-1/mcr-2 genes in the fecal microbiota of Canadian commercial pigs during the growing and finishing period. *VMRR* 10 65–67. 10.2147/VMRR.S202331 31309078 PMC6613599

[B52] Robinson T. P. Wint G. R. W. Conchedda G. Boeckel T. P. V. Ercoli V. Palamara E. (2014). Mapping the Global Distribution of Livestock. *PLoS One* 9:e96084. 10.1371/journal.pone.0096084 24875496 PMC4038494

[B53] Schwarzer G. (2021). *meta-General Package for Meta-Analysis.* Vienna: R Core Team.

[B54] Seemann T. (2021a). *Abricate.* San Francisco, CA: Github.

[B55] Seemann T. (2021b). *Shovill.* San Francisco, CA: Github.

[B56] Slettemeås J. S. Sunde M. Ulstad C. R. Norström M. Wester A. L. Urdahl A. M. (2019). Occurrence and characterization of quinolone resistant *Escherichia coli* from Norwegian turkey meat and complete sequence of an IncX1 plasmid encoding qnrS1. *PLoS One* 14:e0212936. 10.1371/journal.pone.0212936 30856202 PMC6411123

[B57] Torres R. T. Carvalho J. Fernandes J. Palmeira J. D. Cunha M. V. Fonseca C. (2021). Mapping the scientific knowledge of antimicrobial resistance in food-producing animals. *One Health* 13:100324. 10.1016/j.onehlt.2021.100324 34541280 PMC8435696

[B58] Tuszynski J. (2021). *caTools-Tools: Moving Window Statistics, GIF, Base64, ROC AUC, etc.* Vienna: R Core Team.

[B59] Van Boeckel T. P. Brower C. Gilbert M. Grenfell B. T. Levin S. A. Robinson T. P. (2015). Global trends in antimicrobial use in food animals. *Proc. Natl. Acad. Sci.* 112 5649–5654. 10.1073/pnas.1503141112 25792457 PMC4426470

[B60] Van Boeckel T. P. Pires J. Silvester R. Zhao C. Song J. Criscuolo N. G. (2019). Global trends in antimicrobial resistance in animals in low- and middle-income countries. *Science* 365:aaw1944. 10.1126/science.aaw1944 31604207

[B61] Wang Y. Xu C. Zhang R. Chen Y. Shen Y. Hu F. (2020). Changes in colistin resistance and mcr-1 abundance in *Escherichia coli* of animal and human origins following the ban of colistin-positive additives in China: an epidemiological comparative study. *Lancet Infect. Dis.* 20 1161–1171. 10.1016/S1473-3099(20)30149-3 32505232

[B62] Wickham H. Chang W. Henry L. Pedersen T. Takahashi K. Wilke C. (2021). *ggplot2-Create Elegant Data Visualisations Using the Grammar of Graphics.* Vienna: R Core Team.

[B63] Wu R. Yi L. Yu L. Wang J. Liu Y. Chen X. (2018). Fitness Advantage of mcr-1–Bearing IncI2 and IncX4 Plasmids *in Vitro*. *Front. Microbiol.* 9:331. 10.3389/fmicb.2018.00331 29535696 PMC5835064

[B64] Zhou Z. Alikhan N.-F. Mohamed K. Fan Y. the Agama Study Group Achtman M. (2020). The EnteroBase user’s guide, with case studies on *Salmonella* transmissions, *Yersinia pestis* phylogeny, and *Escherichia* core genomic diversity. *Genome Res.* 30 138–152. 10.1101/gr.251678.119 31809257 PMC6961584

[B65] Zingali T. Reid C. J. Chapman T. A. Gaio D. Liu M. Darling A. E. (2020). Whole genome sequencing analysis of porcine faecal commensal *Escherichia coli* carrying class 1 integrons from sows and their offspring. *Microorganisms* 8:microorganisms8060843. 10.3390/microorganisms8060843 32512857 PMC7355456

