# Supplementary material for: Global Distribution of Fluoroquinolone and Colistin Resistance and Associated Resistance Markers in Escherichia coli of Swine Origin – A Systematic Review and Meta-Analysis
Source: Front Microbiol. 2022 Mar 9;13:834793. doi: 10.3389/fmicb.2022.834793 (PMC8961385; doi:10.3389/fmicb.2022.834793)
Supplement: Supplementary File 1 — Database search strings. [file Data_Sheet_1.docx]

**S1- Database search strings**

PubAg- ((antibiotic or drug or antibacterial or antimicrobial or colistin or polymyxin or lactamase or beta or quinolone or fluoroquinolone or carbapenemase) and (resistance or susceptibility) and (pig or sus or porcine or swine) and (escherichia coli or e coli))

Web of Science- ((TS=((antibiotic or drug or antibacterial or antimicrobial or colistin or polymyxin or lactamase or beta or quinolone or fluoroquinolone or carbapenemase) and (resistance or susceptibility) and (pig or sus or porcine or swine) and (escherichia coli or e coli)))) *AND***LANGUAGE:** (English)

PubMed- (("Drug Resistance"[Mesh]) or "Polymyxins"[Mesh]) AND "Swine"[Mesh] AND "Escherichia coli"[Mesh] )

CAB Abstracts- ((antibiotic or drug or antibacterial or antimicrobial or colistin or polymyxin or lactamase or beta or quinolone or fluoroquinolone or carbapenemase or spectrum) and (Pig or Swine or Porcine or Sus) and (resistance or susceptibility or resistant) and (escherichia coli or e coli)).af.
