# Supplementary material for: Global Distribution of Fluoroquinolone and Colistin Resistance and Associated Resistance Markers in Escherichia coli of Swine Origin – A Systematic Review and Meta-Analysis
Source: Front Microbiol. 2022 Mar 9;13:834793. doi: 10.3389/fmicb.2022.834793 (PMC8961385; doi:10.3389/fmicb.2022.834793)
Supplement: Supplementary File 3 — Pooled prevalence estimates of fluoroquinolone and colistin resistance in diseased pigs. [file Data_Sheet_3.docx]

**S3. Pooled prevalences of fluoroquinolone and colistin resistance in diseased pigs**

**Prevalence of fluoroquinolone resistance in isolates from diseased pigs**

The prevalences of fluoroquinolone resistance in isolates from diseased pigs were less than 1% and between 1-10% in 20 and 8 European countries, respectively. Higher levels (10-20%) were reported in Estonia, Austria and Bulgaria, and even higher (25-30%) levels in isolates from Lithuania, Latvia, Serbia and Croatia, while the highest estimates (>44%) were found in isolates from diseased pigs in Spain, Portugal and Romania. Similarly, in isolates collected from diseased pigs, the prevalence of fluoroquinolone resistance remained very low-low (0-2%) in Cuba, Australia and Canada, with the exception of USA where this prevalence was higher (10%).

The prevalences of fluoroquinolone resistance in isolates from diseased pigs ranged between moderate-high (15-40%) in India, Vietnam, Thailand, Japan and South Korea to very high (51-70%) in Taiwan and China. Data from diseased pigs was available from only 3 countries in South America and Africa. The prevalence of fluoroquinolone resistance in isolates from diseased pigs varied between very low (0% in Uganda) to high-very high (36-57% in Brazil and Argentina).


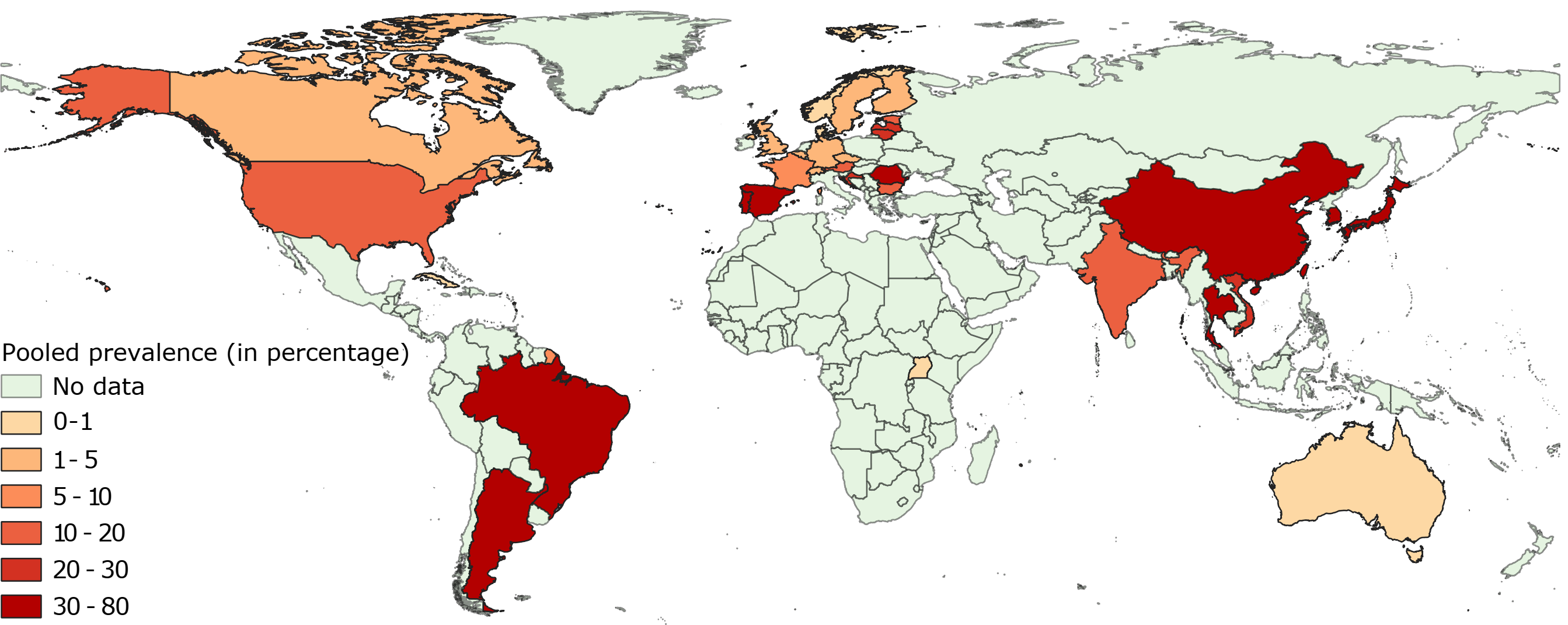


Figure S3a. Global prevalence of fluoroquinolone in isolates from diseased pigs

**Prevalence of colistin resistance in isolates from diseased pigs**

Pooled prevalences of colistin resistance in isolates from diseased pigs in European countries were more variable and ranged between very low (<1% in Finland, Switzerland, Denmark), low (1-10% in Sweden, UK, Estonia, Belgium), moderate (11.5%, Germany) and high (24%, Spain).

Similarly, for isolates from diseased pigs in Asia, prevalence of colistin resistance varied from low (5.7%, South Korea), moderate (17%, China) to high (31-45%, Vietnam, Japan, Taiwan, Thailand). There was only one estimate from South America wherein 6.35% of isolates from diseased pigs were colistin resistant in Brazil.


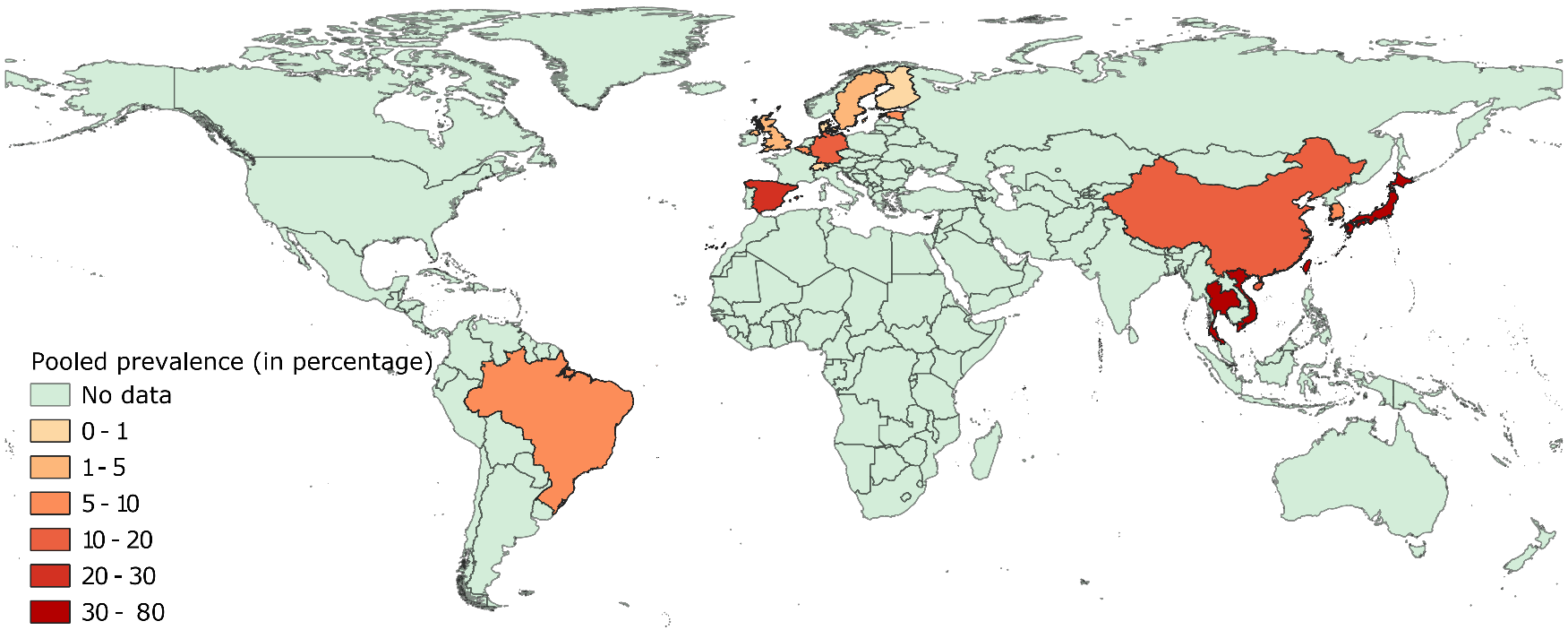


Figure S3b. Global prevalence of colistin resistance in isolates from diseased pigs.

**Statistical heterogeneity in isolates from diseased pigs**

In 50 global estimates of pooled prevalences of fluoroquinolone and colistin resistance in isolates from diseased pigs, 27 (54%) were statistically heterogenous and in 16 cases (32%), heterogeneity was not estimated (Supplementary file S2). Unlike differences in heterogeneity estimates between isolates from healthy pigs collected from European and non-European countries, such intercontinental differences were not observed in heterogeneity estimates from isolates collected from diseased pigs.
