# Supplementary material for: Global Distribution of Fluoroquinolone and Colistin Resistance and Associated Resistance Markers in Escherichia coli of Swine Origin – A Systematic Review and Meta-Analysis
Source: Front Microbiol. 2022 Mar 9;13:834793. doi: 10.3389/fmicb.2022.834793 (PMC8961385; doi:10.3389/fmicb.2022.834793)
Supplement: Supplementary File 4 — Prevalence of mcr and qnr genes in articles selected for the literature review. [file Data_Sheet_4.docx]

**S4. Prevalence of *mcr* and *qnr* genes in articles selected for the literature review**

**Prevalence of *mcr* genes-**

*mcr-1* was the most prevalent and commonly studied colistin resistance gene, with information available from four continents. This gene was most common in isolates from Asia, where a prevalence of 13.1% and 16.2% was found in isolates from healthy and diseased pigs in Asia, respectively. In Europe, prevalence of this gene was 12% in isolates from diseased pigs but low (0.43%) in isolates from healthy pigs. Prevalence of this gene was 0.36% and 5.88% in isolates from healthy pigs in North and South America, respectively.

Other prevalent *mcr* genes in isolates from Asia were *mcr-5* (4.35%- diseased pigs, 0%- healthy pigs), *mcr-9* (2.57%- healthy pigs) and *mcr-3* (2.3%- healthy pigs, 0.95%- diseased pigs). The remaining mcr genes described in the literature (*mcr-2, mcr-4, mcr-6, mcr-7, mcr-8*) were either not detected or their prevalence was below 0.39% across different sources. In European isolates, prevalence of other *mcr* genes was 0-1% with the exception of *mcr-4* which was present in 17.5% of isolates from diseased pigs. No data was available for prevalence of genes other than *mcr-1* in North and South American isolates.

**Prevalence of *qnr* genes-**

*qnrS* was by far the most prevalent *qnr* gene in isolates from Asia (12.8-healthy pigs, 13.2%- diseased pigs), followed by *qnrD* (3%-healthy pigs, 0%- diseased pigs), *qnrB* (1.43%- diseased pigs, 0.68%- healthy pigs) and *qnrA* (1.05%- healthy pigs, 0.08%- diseased pigs). *qnrC* was not detected in any Asian isolates. In contrast, *qnrB* was the most prevalent gene in North American isolates (2.54%- healthy pigs, 1.09%- diseased pigs) followed by *qnrS* (2.03%- healthy pigs). Prevalence of *qnr* genes in European isolates was extremely low (0-0.07%. in healthy pigs).

Pooled prevalence of *mcr* and *qnr* genes in swine *E. coli* isolates at continent-level (with 95% confidence intervals)

| Health Status | Gene | Asia | Europe | North America | South America |
| --- | --- | --- | --- | --- | --- |
| Healthy | *mcr-1* | 13.07 (8.2-18.83) | 0.43 (0-1.31) | 0.36 (0.01-1.07) | 5.88 (0-23.54) |
|  | *mcr-2* | 0.4 (0-3.37) | 0 (0-0.1) | - | - |
|  | *mcr-3* | 2.7 (0.46-6.25) | 0 (0-7.43) | - | - |
|  | *mcr-4* | 0 (0-0.04) | 0 (0-7.43) | - | - |
|  | *mcr-5* | 0 (0-3.65) | 0 (0-7.43) | - | - |
|  | *mcr-6* | 0.39 (0.05-0.97) | - | - | - |
|  | *mcr-7* | 0.39 (0.05-0.97) | - | - | - |
|  | *mcr-8* | 0.13 (0-0.55) | - | - | - |
|  | *mcr-9* | 2.57 (1.56-3.81) | - | - | - |
| Diseased | *mcr-1* | 16.21 (7.67-27.04) | 11.99 (1.65-29.34) | - | - |
|  | *mcr-2* | 0 (0-0) | 0.14 (0-2.11) | - | - |
|  | *mcr-3* | 0.95 (0.02-2.73) | 0 (0-0.02) | - | - |
|  | *mcr-4* | 0 (0-0.05) | 17.46 (0-61.02) | - | - |
|  | *mcr-5* | 4.35 (0-67.26) | 0.91 (0-3.51) | - | - |
|  | *mcr-6* | 0 (0-1.21) | - | - | - |
|  | *mcr-7* | 0 (0-1.21) | - | - | - |
|  | *mcr-8* | 0 (0-1.21) | - | - | - |
| Healthy | *qnrA* | 1.05 (0-3.94) | 0 (0-6.27) | - | - |
|  | *qnrB* | 0.68 (0-2.94) | 0 (0-6.27) | 2.54 (1.18-4.36) | - |
|  | *qnrC* | 0 (0-0.05) | - | - | - |
|  | *qnrD* | 3.02 (0-12.76) | - | - | - |
|  | *qnrS* | 12.81 (5.03-23.16) | 0.07 (0-0.68) | 2.03 (0.83-3.7) | - |
| Diseased | *qnrA* | 0.08 (0-0.57) | - | - | - |
|  | *qnrB* | 1.43 (0.04-4.1) | - | 1.09 (0.02-3.27) | - |
|  | *qnrC* | 0 (0-0) | - | - | - |
|  | *qnrD* | 0 (0-0) | - | - | - |
|  | *qnrS* | 13.23 (5.48-23.52) | - | - | - |
